# Influence of Ti Content on the Partial Oxidation of Ti_x_FeCoNi Thin Films in Vacuum Annealing

**DOI:** 10.3390/ma10101141

**Published:** 2017-09-27

**Authors:** Ya-Chu Yang, Jien-Wei Yeh, Chun-Huei Tsau

**Affiliations:** 1 Department of Materials Science and Engineering, National Tsing Hua University, Hsinchu 300, Taiwan; yachu.y@gmail.com; 2 Institute of Nanomaterials, Chinese Culture University, Taipei 111, Taiwan

**Keywords:** sputtering, vacuum annealing, oxide, thin films

## Abstract

This study investigated the effects of Ti content and vacuum annealing on the microstructure evolution of Ti_x_FeCoNi (x = 0, 0.5, and 1) thin films and the underlying mechanisms. The as-deposited thin film transformed from an FCC (face center cubic) structure at x = 0 into an amorphous structure at x = 1, which can be explained by determining topological instability and a hard ball model. After annealing was performed at 1000 °C for 30 min, the films presented a layered structure comprising metal solid solutions and oxygen-deficient oxides, which can be major attributed to oxygen traces in the vacuum furnace. Different Ti contents provided various phase separation and layered structures. The underlying mechanism is mainly related to the competition among possible oxides in terms of free energy production at 1000 °C.

## 1. Introduction

A very low resistivity (30 μΩ·cm) of the TiFeCoNiO_x_ thin film is observed in our previous study [1], and it is observed during the test of oxidation resistance of the thin films. This phenomenon is caused by the different activities with oxygen, Ti has higher affinity with oxygen than the other elements. Therefore, Ti migrates to the surface and forms a TiO-rich oxide, the other elements form a FeCoNi-rich alloy which provides a very good conductivity. A similar phenomenon is also observed in AlFeCoNiO_x_ oxide film, because aluminum also has a very high affinity with oxygen [2]. However, the resistivity of CrFeCoNiO_x_ oxide film is not as low as the oxide films of TiFeCoNiO_x_ and AlFeCoNiO_x_ because of its activity with oxygen. Also, the lowest resistivity of every oxide film is achieved after 1000 °C for 30 min vacuum annealing; after that, the resistivity increases with increasing the annealing time. This decreasing of resistivity of these thin films contributes to the recrystallization and deficient oxidation. This phenomenon is very interesting for academic research.

The parent study investigated the phase transformations and deficient oxides through vacuum annealing. The mechanism of partial oxidation is an important factor to understand the origin of low resistivity. In this study, thin films with various quantities of Ti, such as Ti_x_FeCoNi (x = 0, 0.5, 1.0), were designed and prepared through sputter deposition and subsequent vacuum annealing to reveal the mechanism of the partial oxidation of Ti_x_FeCoNi film during vacuum annealing.

## 2. Experimental Procedures

FeCoNi (designated as Ti_0_), Ti_0.5_FeCoNi (Ti_0.5_), and TiFeCoNi (Ti_1.0_) alloy targets with a diameter of 2 inches were prepared from high-purity Ti, Fe, Co, and Ni via vacuum arc melting and machining. The chemical compositions of the Ti_x_FeCoNi targets listed in Table 1 were determined using an energy dispersive spectrometer (EDS) of a field emission scanning electron microscope (SEM, JEOL JSM-6335, JEOL Ltd., Tokyo, Japan) operated at 15 kV. The substrate used for deposition was a SiO_2_/Si wafer, whose 0.3 μm thick SiO_2_ layer was formed by heat treatment at 1000 °C for 24 h. The SiO_2_ layer served as a barrier to prevent the diffusion of Si from the substrate to the film during high-temperature annealing. Ti_x_FeCoNi thin films were deposited on the SiO_2_/Si substrates by using a direct current (DC) sputtering system, without bias and temperature control. The substrates were placed approximately 11 cm from the target on the center of a substrate table which was rotated at a speed of 5 rpm. Prior to deposition, both the substrate and target were independently sputter cleaned by means of a shutter placed between them. It was performed at 100 W and the flow rate of Ar was 30 standard cubic centimeter per min (sccm). The background and working pressures were 5 × 10^−5^ and 2 × 10^−3^ torr, respectively. The deposition power was 100 W and the deposition rate was 133 Å/min. Some of the deposited samples were further annealed in a vacuum tube furnace at 1000 °C for 30 min.

The crystallographic structures of the Ti_x_FeCoNi films were examined using glancing angle incidence (2°) X-ray diffractometer (XRD, Rigaku TTRAX III, Rigagu Ltd., Tokyo, Japan) with Cu Kα_1_ radiation (λ = 0.15406 nm) generated at 50 kV and 300 mA. The microstructures and compositions of the thin films were further investigated in detail by utilizing a field emission transmission electron microscope (TEM, FEI Tecnai S-Twin, Thermo Fisher Scientific, Waltham, MA, USA) equipped with an EDS.

## 3. Results and Discussion

**Figure 1:**
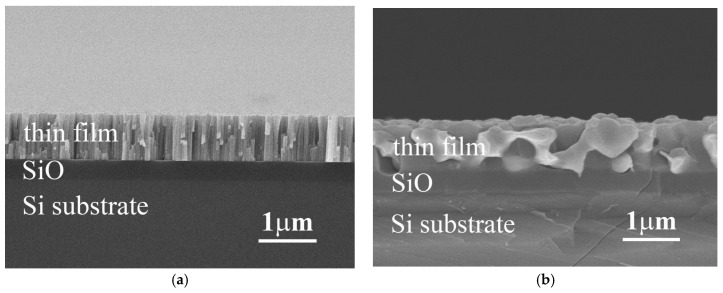
The typical SEM micrographs of (**a**) the as-deposited Ti_0_ thin film; and (**b**) the as-annealed Ti_0.5_ thin film.

Figure 1a,b show the typical cross-section SEM images of as-deposited and as-annealed Ti_x_FeCoNi thin films, respectively. All of these three alloy thin films had a similar micrograph under as-deposited or as-annealed state. The as-deposited thin film has a columnar structure, shown in Figure 1a. However, each single column was not a single grain. TEM observation described below proved that the FeCoNi alloy thin film had a nano-grained structure. This nano-grained structure would become an amorphous structure after the Ti-content increased; the TiFeCoNi alloy thin film thus had a fully amorphous structure. The microstructures of these thin films become to a coarse-grained one after vacuum annealing at 1000 °C for 30 min, shown in Figure 1b; and different phases were formed after annealing because of diffusion of atoms and oxidation.

**Figure 2:**
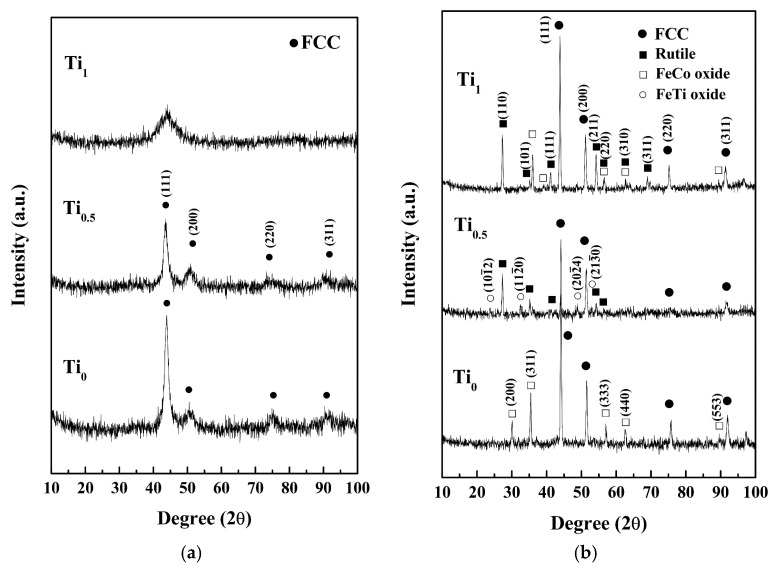
Glancing incident angle XRD patterns of (**a**) as-deposited; and (**b**) as-annealed Ti_x_FeCoNi films.

Figure 2 presents the XRD patterns and the crystallographic structures of Ti_x_FeCoNi thin films in as-deposited and as-annealed states. The FCC peaks indicate that the as-deposited Ti_0_ and Ti_0.5_ films present a single FCC structure with a nano-crystalline structure, which is confirmed in the latter section of the TEM analysis; while the broad peak shows that the Ti_1.0_ film has an amorphous structure. The formation of an FCC structure through the as-deposited Ti_0_ film is reasonable because Fe, Co, and Ni atoms can substitute one another due to their similar atomic sizes, valences, and electronegativities [3]. Intensity decreases with Ti content because of the large atomic size of Ti. Thus, large lattice distortion and subsequent diffuse scattering are induced. An increase in the half-height width of the main FCC peak corresponds to a decrease in grain size that can be attributed to the reduced atom migration and grain growth when large Ti atoms are incorporated. The amorphous structure of the Ti_1.0_ film can be explained by the hard ball model proposed by Kao et al. [4]. The radii of Fe, Co, Ni, and Ti are 1.27, 1.25, 1.25, and 1.46 Å, respectively [3]. The average atomic radius of Ti_1.0_ is 1.3075 Å, and the atomic size fluctuation is between +11.7% and −4.4%. An amorphous structure is expected because the low size fluctuation of −4.4% does not satisfy the least size deviation requirement of ±7.2% for the merging of the second and third atomic shells for a short-range-order amorphous structure and ±6.2% for the merging of the fourth and fifth shells for a medium-range-order amorphous one. Table 2 lists the chemical compositions of the as-deposited thin films analyzed by SEM/EDS. Also all of the as-deposited thin films contained 7–8 at % oxygen which was from the deposition process.

Figure 2b reveals new diffraction peaks in the XRD patterns of the films subjected to vacuum annealing. The Ti_0_ film presents the FeCoNi peaks and FeCo-rich oxide peaks that correspond to the FCC structures with lattice constants of 3.55 and 8.38 Å, respectively. The Ti_0.5_ film yields FeCoNi-rich, rutile-TiO_x_, and FeTi-rich oxide peaks. FeCoNi-rich peaks show an FCC structure with a lattice constant of 3.59 Å. Rutile peaks have a TiO_x_ structure (JCPDS: 76-0321) with lattice constants of *a* = 4.59 Å and *c* = 2.95 Å. FeTi-rich oxide peaks have a HCP structure with lattice constants of *a* = 5.09 Å and *c* = 14.06 Å, which are similar to FeTiO_3_ (JCPDS: 79-1838). The Ti_1.0_ film presents FeCoNi-rich, FeCo-rich oxide, and rutile peaks. The FeCoNi-rich and FeCo-rich oxide peaks indicate an FCC structure with lattice constants of 3.57 and 8.75 Å, respectively. However, the rutile peaks correspond to the TiO_x_ structure (JCPDS: 76-0321) with lattice constants of *a* = 4.609 Å and *c* = 2.963 Å. Overall, the as-annealed thin films retained an FCC and FeCoNi-based metal phase and formed oxides.

**Figure 3:**
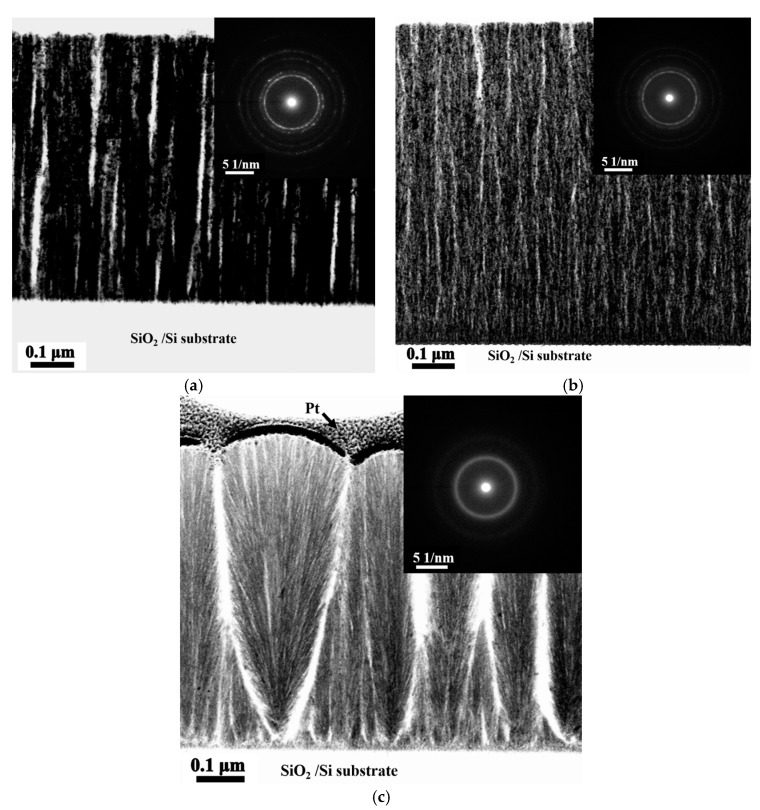
TEM BF images and corresponding SAD of as-deposited Ti_x_FeCoNi thin films: (**a**) Ti_0_ film; (**b**) Ti_0.5_ film; and (**c**) Ti_1.0_ film.

Figure 3a–c present the TEM bright field (BF) images and the corresponding selection area diffraction patterns (SAD) of the cross-sectional microstructures of the as-deposited Ti_0_, Ti_0.5_, and Ti_1.0_ thin films with a columnar structure and void striations (white area) along the column boundaries [5]. The voids were unavoidable because of the shadow effect associated with oblique deposition at room temperature and without an applied bias voltage. Without atom mobility and ion bombardment enhanced by high temperature and bias bombardment, eliminating the formation of voids during deposition is difficult. Additionally, the ring patterns of these thin films indicates that the Ti_0_ and Ti_0.5_ alloy thin films have nano-crystalline structures, and Ti1 alloy thin film has an amorphous structure.

**Figure 4:**
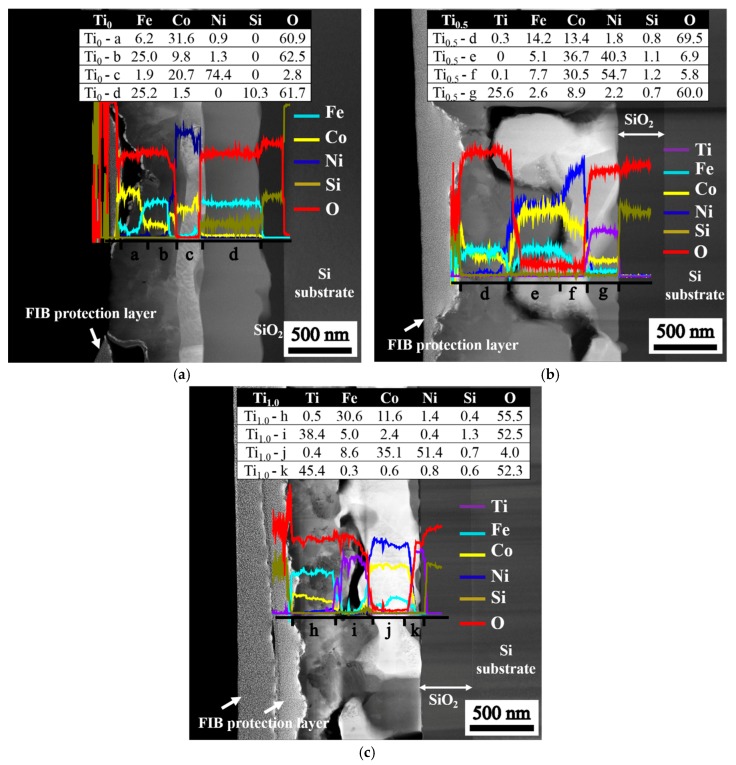
Composition distribution of as-annealed Ti_x_FeCoNi thin films, as determined using TEM and EDS: (**a**) Ti_0_; (**b**) Ti_0.5_; and (**c**) Ti_1.0_ films.

Figure 4 illustrates the structure of stacked layers after annealing is conducted at 1000 °C for 30 min. The variations in the composition along the vertical axis (through thickness) of the layered structures are revealed by the line scan. The compositions at the four selected positions from the surface to the SiO_2_ interfacial layer are listed in the inserted tables, and the composition sequences for different films are as follows: (1) Ti_0_ film, Fe_6.6_Co_31.6_Ni_0.9_O_60.9_ → Fe_26.4_Co_9.8_Ni_1.3_O_62.5_ → Fe_2.0_Ni_20.7_Co_74.4_O_2.9_ → Fe_26.8_Co_1.7_Si_10.1_O_61.4_; (2) Ti_0.5_ film, Ti_0.3_Fe_14.3_Co_13.5_Ni_1.6_Si_0.7_O_69.6_ → Fe_15.1_Ni_36.7_Co_40.3_Si_1.1_O_6.8_ → Ti_0.1_Fe_7.8_Ni_30.7_Co_54.5_Si_1.2_O_5.7_ → Ti_25.8_Fe_2.6_Co_8.9_Ni_2.2_Si_0.6_O_59.9_; and (3) Ti_1.0_ film, Ti_0.5_Fe_30.6_Co_11.6_Ni_1.4_Si_0.4_O_55.5_ → Ti_39.3_Fe_4.8_Co_0.5_Ni_0.4_Si_1.3_O_53.7_ → Ti_0.3_Fe_8.7_Co_35.3_Ni_51.8_Si_0.6_O_3.3_ → Ti_45.4_Fe_0.3_Co_0.6_Ni_0.8_Si_0.6_O_52.3_. The oxygen content of the fully-oxidized Me_2_O_3_ (60 at % O) and MeO_2_ (66.67 at % O) suggests that some of the oxides that form with the residual FeCoNi-rich metal are deficient in oxygen because the oxygen source is insufficient to oxidize the films completely. This oxygen deficiency accounts for the high conductivity of the as-annealed films because more oxygen vacancies can provide more electron carriers to enhance electrical conductivity.

**Figure 5:**
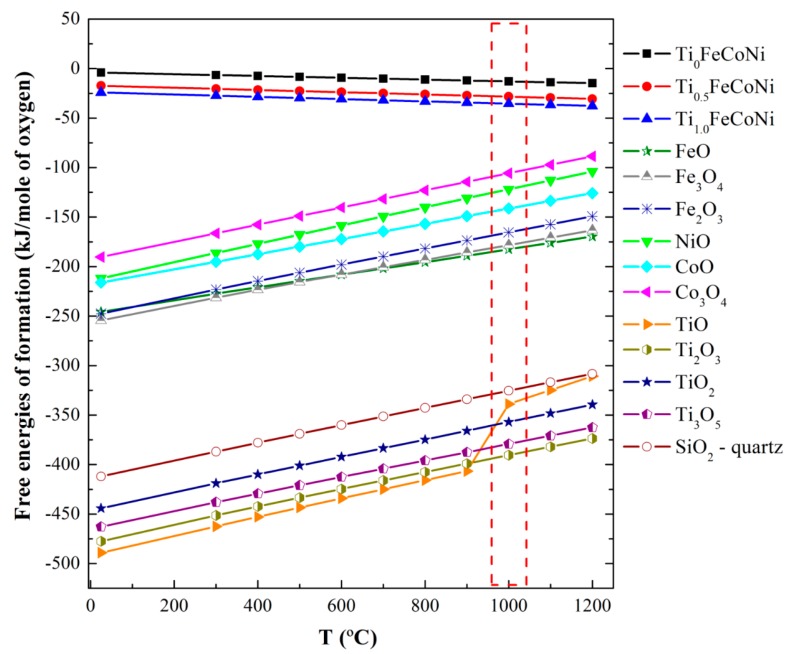
Free energies involved in the formation of Ti*_x_*FeCoNi metal and A_n_O_m_ (A = Ti, Fe, Co, Ni, and Si) oxides.

The layered structure with different phases can be explained from the perspectives of thermodynamics and kinetics. The formation of this structure can be attributed to two factors. (1) Chemical affinity competition or stronger affinity in metal–oxygen pairs, such as SiO and TiO, is preferred to form oxides [6,7]. At 1000 °C, the free energies of the formation follow the increasing order of TiO < SiO < FeO < CoO < NiO (Figure 5); (2) Diffusion rate competition due to the decreasing order of O > Fe > Co > Ni > Ti [8] involves the formation of Fe and Co atoms with high diffusion rates on the top layer, although Ti atoms likely form oxides. The free energies in Figure 5 have been calculated at different temperatures by using Equations (1)–(3) [7]

Δ*H_t_* = Δ*H*_0_ + 2.303*aT logT* + *b ×* 10^−3^*T*^2^ + *c ×* 10^5^*T*^−1^ (1)

Δ*S_t_* = −*a* − 2.303*aT logT* − 2*b ×* 10^−3^*T* + *c ×* 10^5^*T*^−2^ − *I* (2)

Δ*F_t_* = Δ*H* − *T*Δ*S* (3)

where Δ*F_t_* is the free energy of formation, Δ*S_t_* is the entropy of formation, Δ*H* is the enthalpy of formation, and *a*, *b*, and *I* are constants [7].

**Figure 6:**
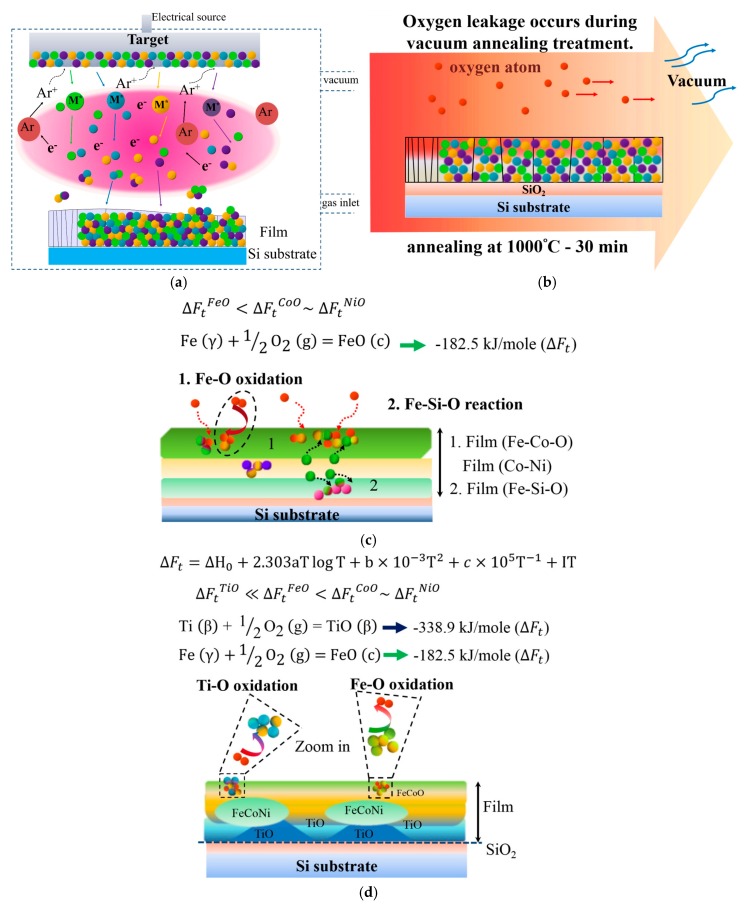
Schematic diagram showing the formation of phases in Ti_x_FeCoNi films from the as-deposited state to the post-annealed state: (**a**) DC sputtering deposition of thin film on Si substrate; (**b**) oxygen coming from the vacuum chamber and SiO_2_ substrate during vacuum annealing; (**c**) diffusion pathways and layer formation in Ti_0_ film during annealing; and (**d**) diffusion pathways and layer formation in Ti_0.5_ and Ti_1.0_ films during annealing.

Figure 6 presents a schematic of atom migration, diffusion pathways, and reactions during annealing to explain the formation of the layered structure. Figure 6a illustrates the deposition of Ti_x_FeCoNi thin films via DC sputtering, resulting in the uniform deposition of constituent atoms on the SiO_2_/Si substrate. Figure 6b shows that oxygen transfers from the chamber at a pressure of 1 × 10^−2^ torr and from the SiO_2_/Si substrate under heat treatment at 1000 °C for 30 min. Figure 6c displays the Ti_0_ thin film, in which Fe and Co atoms preferentially react with oxygen to form the top FeCo-rich oxide layer, and with oxygen from the interface layer adjacent to the SiO_2_ to form FeSi-rich oxide layers. Fe–O is even stronger than Co–O. Thus, FeCoNi-rich solid solution phase (Ni > Co > Fe in concentration) formed as the middle layer. By contrast, the bonding energy of Ti–O is stronger than those of Ti–Fe, Ti–Co, Ti–Ni, Fe–O, Ni–O, and Co–O in the Ti-containing thin film. Figure 6d shows that this strong affinity causes Ti atoms to migrate into the oxygen-rich region of the thin films during annealing. Thus, the Ti oxide Ti_25.8_Fe_2.6_Co_8.9_Ni_2.2_Si_0.6_O_59.9_ forms near the SiO_2_ layer in the Ti_0.5_ thin film. However, the Ti and Fe contents in the as-deposited Ti_0.5_ film are 14.3 and 28.6 at %, respectively. The high concentration of Fe (2.6 at %) in Ti oxide suggests that the second-strongest Fe–O bonding can compete with Ti for oxygen when Fe content is relatively high. The FeCoNi-rich metal phase with a large depletion of Ti develops in the middle (see compositions at f and g in Figure 4b). For the Ti_1.0_ film, in which the Ti content is twice as that of the Ti_0.5_ film, TiFe-rich oxide is further produced with FeCo-rich oxide on the top layer (at i and j in Figure 4c). In addition, nearly pure Ti oxide with high Ti content but negligible Fe content (0.3 at %) grows with the Ti-depleted and FeCoNi-rich metal phase at the bottom (at k in Figure 4c).

## 4. Conclusions

The influence of Ti content and vacuum annealing on the microstructure of Ti_x_FeCoNi thin films was investigated. The as-deposited Ti_0_ and Ti_0.5_ films presented a nano-crystalline FCC structure, while the as-deposited Ti_1.0_ possessed an X-ray amorphous structure, which can be explained by determining topological instability and by using a hard ball model. The films comprised a layered structure because of phase separation into metal solid solutions and oxygen-deficient oxides after vacuum annealing at 1000 °C for 30 min. Different Ti contents provided various phase separation and layered structures. The mechanism was mainly related to the competition among possible oxides in terms of free energy production at 1000 °C.

## Figures and Tables

**Table 1:** Chemical compositions of the Ti_x_FeCoNi targets analyzed by SEM/EDS.

Targets	Compositions (at %)			
	Ti	Fe	Co	Ni
Ti_0_	-	32	34	34
Ti_0.5_	12	28	30	30
Ti_1_	23	25	26	26

**Table 2:** Chemical compositions of as-deposited Ti_x_FeCoNi thin films (SEM/EDS).

Thin Film	Composition (at %)				
	O	Ti	Fe	Co	Ni
Ti_0_	7 ± 1	-	27 ± 2	33 ± 1	32 ± 1
Ti_0.5_	7 ± 1	11 ± 1	25 ± 1	30 ± 1	27 ± 1
Ti_1_	9 ± 1	22 ± 1	23 ± 1	25 ± 1	21 ± 1
